# 7 years-delayed presentation of a traumatic diaphragmatic rupture: laparoscopic repair

**DOI:** 10.1186/1471-2482-13-S1-A2

**Published:** 2013-09-16

**Authors:** Giovanni Aprea, Alfonso Canfora, Antonio Ferronetti, Antonio Giugliano, Francesco Guida, Melania Battaglini Ciciriello, Antonio Savanelli, Bruno Amato

**Affiliations:** 1 Department of Gastroenterology, Endocrinology and Surgery, General Surgery Division, University “Federico II” of Naples, Via Pansini, 5, 80131, Naples, Italy

## Summary

Post-traumatic diaphragmatic hernias(PDH) are possible complications of blunt and penetrating thoracic or abdominal trauma. These hernias may be diagnosed at the time of the initial trauma, but are sometimes recognized only after several months or years during examinations for their related symptoms. We here present the case of a patient in which diagnosis was obtained only after 7 years from the accident and for which a successful laparoscopic repair of the hernia was performed.

## Introduction

Traumatic diaphragmatic rupture is a possible life-threatening condition that occurs up to 5% of major thoraco-abdominal traumas. This kind of injury is sometimes diagnosed at the time of the initial trauma referral due to its acute presentation, but sometimes it can escape detection, especially if occurring as an isolated injury. Symptoms such as dyspnea, non-cardiac chest pain, and vasovagal symptoms may start the workup, but PDHs are sometimes discovered incidentally in apparent complete wellness. We here report the case of a massive PDH discovered incidentally during examination for an apparently not related condition.

## Case report

**Figure 1:**
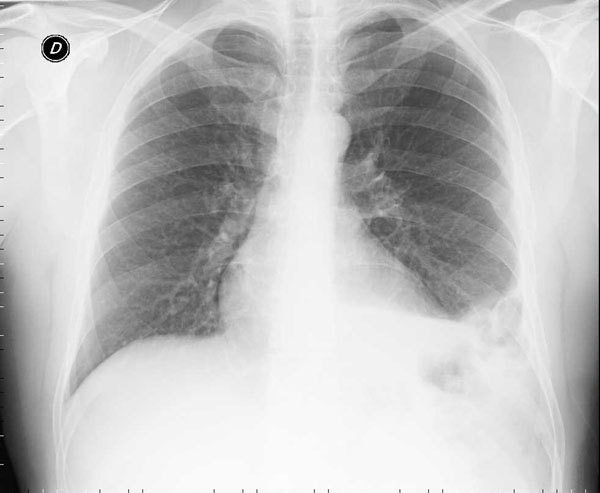
pre-operative chest X-ray

**Figure 2:**
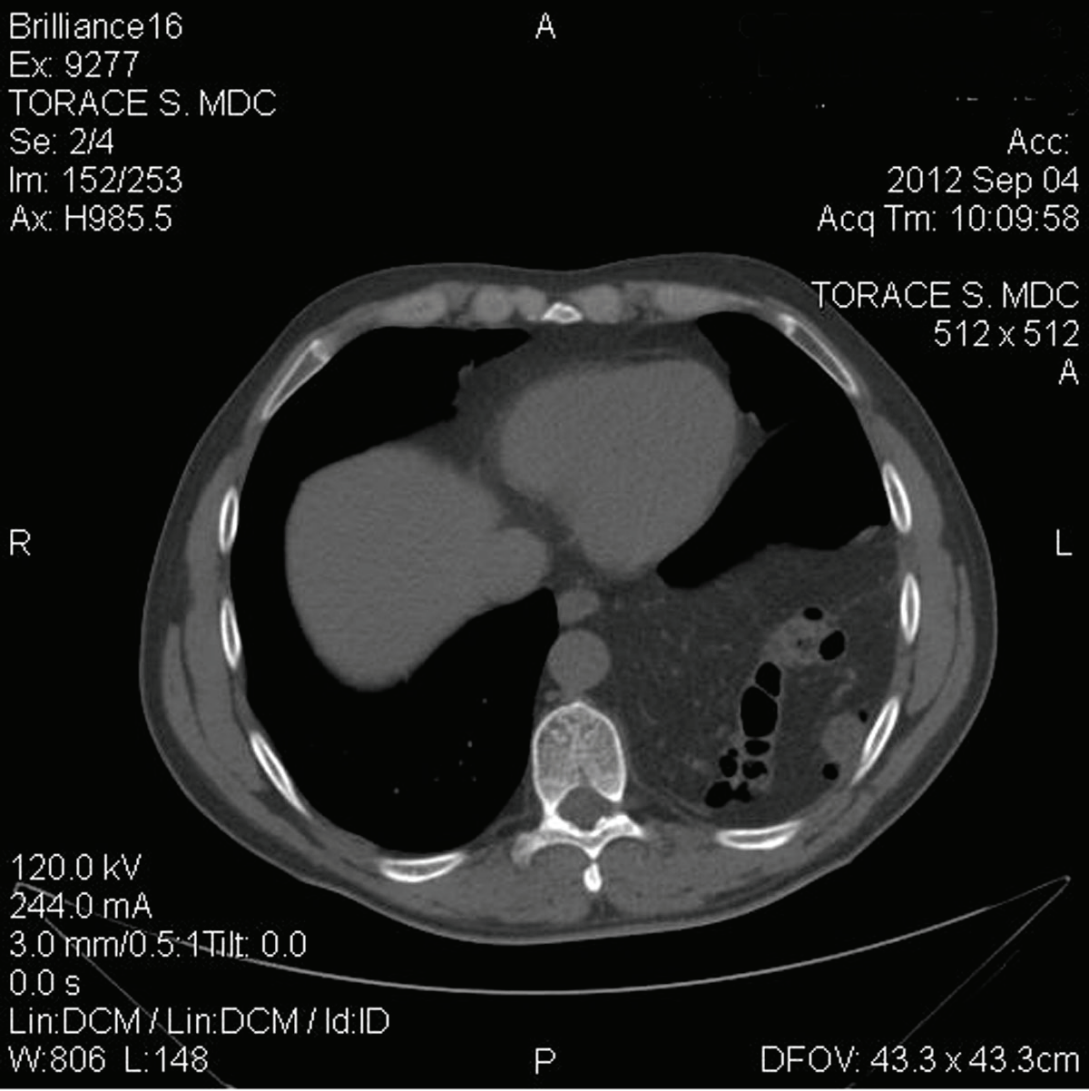
Pre-operative CT-scan

A 70-year old male was involved in a motorcycle accident in the year 2005. After the trauma protocol examination in the emergency unit, the patient was dismissed with no reported damage to the diaphragm or any other organ. The patient reported no symptoms over the next 7 years. In September 2012, due to the presentation of dyspnea after climbing the stairs, he performed a chest x-ray and discovered the presence of a massive left diaphragmatic hernia with dislocation of the colon in the thorax (Figure 1); only after a precise anamnestic investigation the patient admitted a change in his intestinal transit with an evolution to constipation and related chest pain. The patient then practiced a computed tomography (CT) scan that demonstrated a voluminous left diaphragmatic hernia with great part of the left hemithorax occupied by abdominal fat and intestinal loops (left colic flexure and descending colon) (Figure 2). The fat reached and passed an axial plan containing the aortic arch; consensual pulmonary atelectasy was also present. The left postero-lateral defect of the diaphragm had a major axis of 54mms; signs of past rib fractures were present.

**Figure 3:**
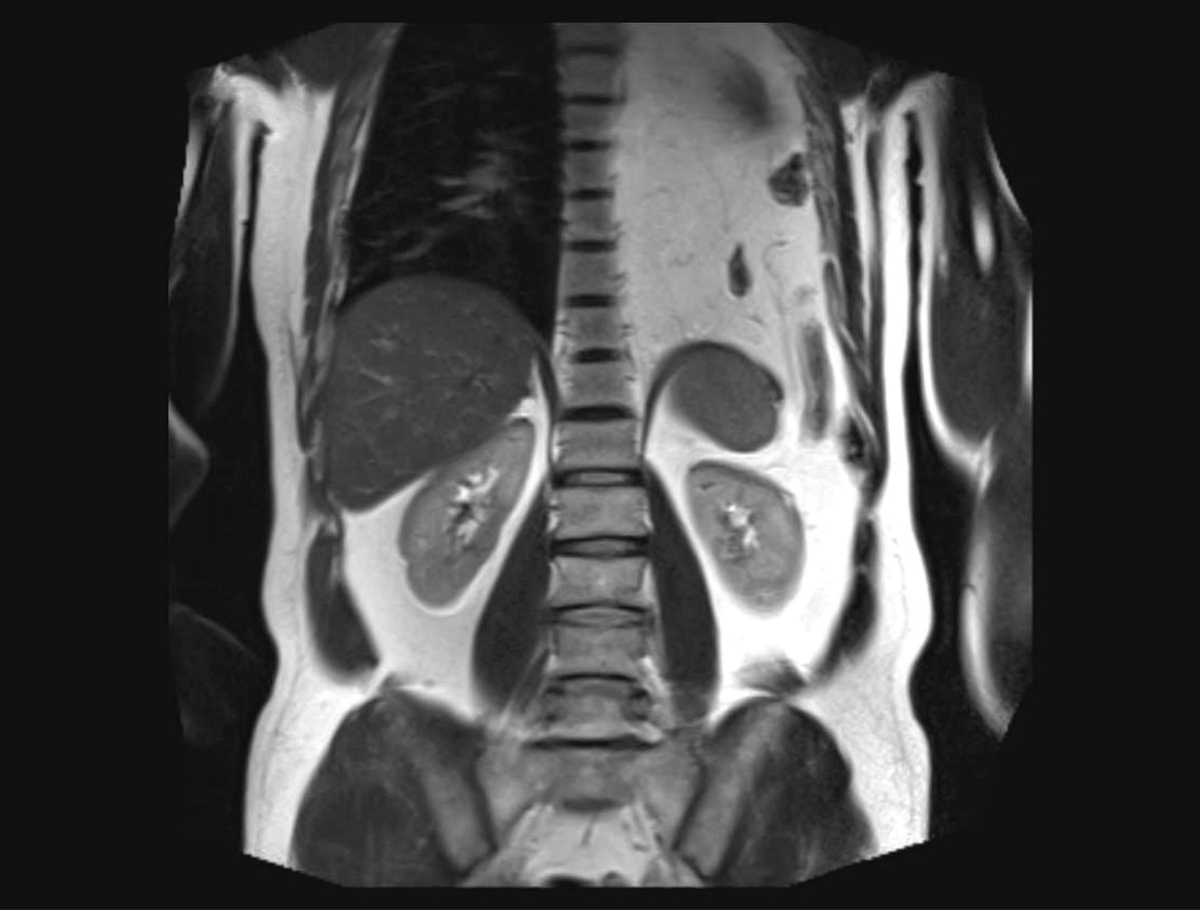
Pre-operative RMI

On the 26^th^ October 2012 the patient was admitted to our tertiary hospital. Routine preoperative testings(laboratory and ECG) didn’t show any abnormal values. A preoperative spirometryand hemogasanalysis was requested by the anesthesiologist:the exams showed normal values (only FEF50% and FEF75% were lightly reduced). To complete the assessment of the diaphragmatic defect an RM scan was performed (Figure 3); the post-traumatic diaphragmatic defect was confirmed in dimensions but was characterized by close relations with the spleen that had been dislocated behind the stomach.

**Figure 4:**
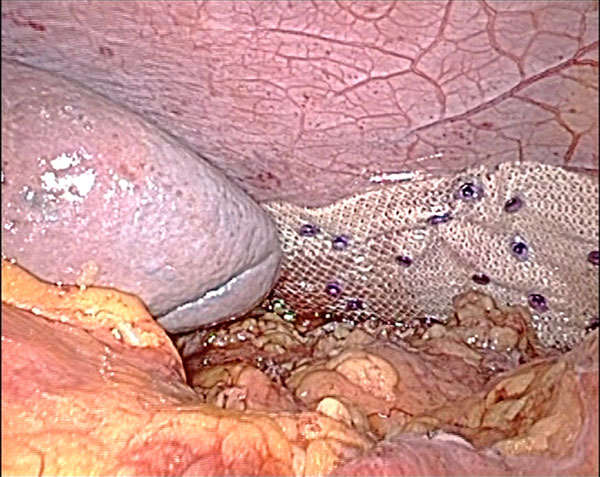
**Laparoscopic repair** The appearance of the repaired defect with Parietex Composite mesh and Absorbatacks.

Due to the possible life-threatening complications for this type of hernia, operative repair was recommended. The patient underwent exploratory laparoscopy on the 6^th^ November 2012. The descending colon, the left colic flexure and the greater omentum were herniated through the diaphragmatic tear. The spleen had intimate relations with the tear. After reduction of the hernia content, a large defect of the left diaphragm was observable. Due to its large size we decided to discard the primary closure option. After an accurate adhesiolysis, the defect was reduced with a few non absorbable separate suturesand bridged with a 10x15 cm Parietex Composite mesh (Covidien. All rights reserved). The mesh was blocked with absorbable tacks (AbsorbaTack™ 5mm.Covidien. All rights reserved). Two 12 mm trocars and three 5mm trocars were necessary to complete the procedure (12-sovraombelicale e ipocondriosn, 5-ipocondrio dx, sottoxifoideo e f.iliacasn.); duration 3 hr. (Figure 4).

**Figure 5:**
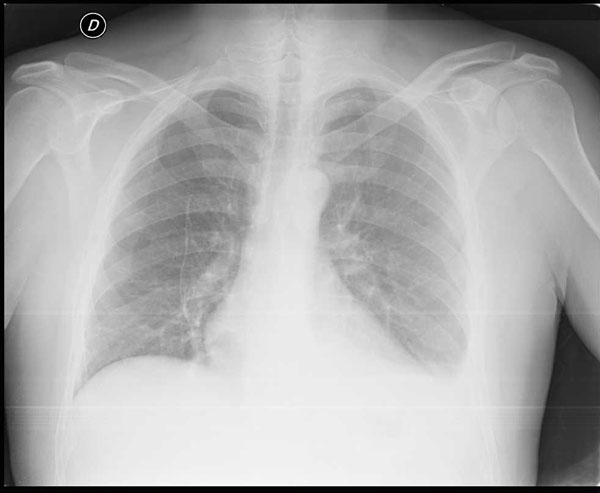
Post-operative chest X-ray

**Figure 6:**
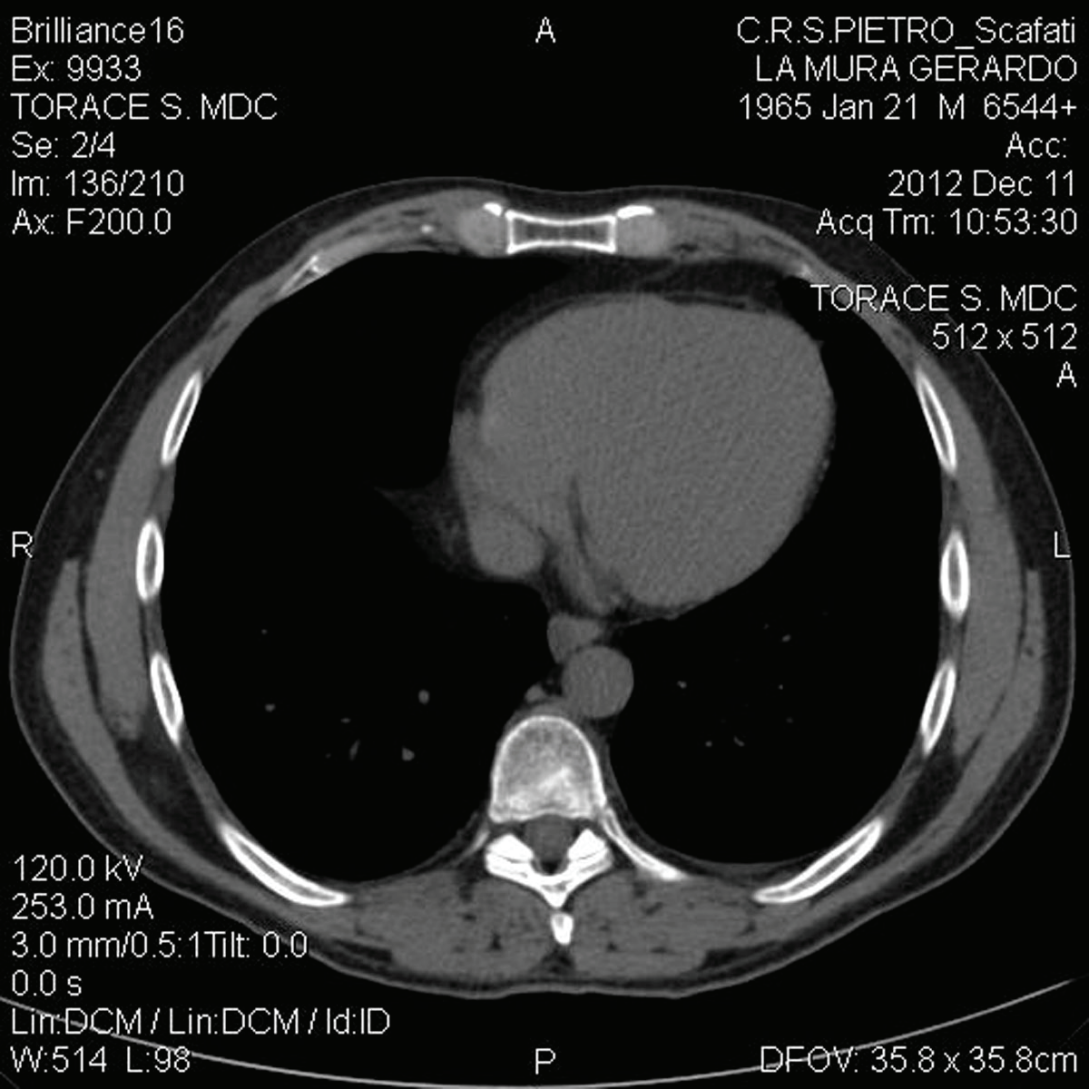
Post-operative CT scan

There was no early postoperative morbidity but we preferred to transfer the patient in the resuscitation unit for a 24 hr-observation.The patient was retransferred to our surgery unit after controlled extubation and a postoperative chest x-ray (Figure 5). The patient was discharged from the hospital on the sixth postoperative day and reports no complications up to now. A postoperative CT scan was performed 1 month after the operation to check the results (Figure 6).

## Discussion

Traumatic diaphragmatic rupturecan go unrecognized at the initial injury and present, in adelayed case, months or even years after the event. Left-sided diaphragmatic ruptures occur three timesmore frequently than right-sided ruptures, since the leftdiaphragm is structurally weaker, as it originates from thepleuro-peritoneal membrane. Right-sided rupture is seen lessfrequently because of the buffering effect of the liver. There is no radiologic gold standard to diagnose a traumaticdiaphragmatic rupture. Chest X-rays may show obliterationof the diaphragmatic shadow or elevation of the diaphragm, but up to 50% of the initial chest X-rayscan be non-diagnostic. CT scan is the preferred diagnosticmodality in cases of suspected diaphragmatic rupture with a 61% sensitivity and 87% specificity. Other diagnostic techniques suchas ultrasound and upper gastrointestinal (GI) contrast studyare not used routinely.

The first successful diaphragmatic repair wasreported by Riolfi in 1886. The surgical treatmentincludes hernia reduction, pleural drainage, and repair of thediaphragmatic tear. Diaphragmatic repair may be performedeither via laparotomy or thoracotomy or via laparoscopy orthoracoscopy. Due to the hemodynamic and respiratory stability of the patient we preferredlaparoscopy for the repair of the diaphragmaticrupture in the presented case. Most authors recommendclosing of the diaphragmatic defect with non-absorbablesutures or with a patch for large defects. We chose a combined approach to the diaphragmatic rupture( sutures plus mesh);no pleural drainage was applied.

## Conclusion

Diaphragmatic rupture may be a not very uncommon complication of significant thoraco-abdominal trauma. Clinical presentation may be subtle, delayedand non-specific. Although plain chest radiography may be helpful inestablishing diagnosis in most cases, computedtomography (CT) is a better diagnostic choice; MRI may add important details of the diaphragmatic defect. Thepotential life-threatening complications of massive diaphragmatic hernia mandate a promptrepair. A trans-abdominal approach is preferred for surgicalclosure, as it provides good access to the tear in the diaphragm. The treatment consists of closing the defect withnon-absorbable sutures or a patch. Our experience demonstrates laparoscopy as a safe procedure.
